# Loss of the Osteogenic Differentiation Potential during Senescence Is Limited to Bone Progenitor Cells and Is Dependent on p53

**DOI:** 10.1371/journal.pone.0073206

**Published:** 2013-08-29

**Authors:** Geneviève Despars, Cynthia L. Carbonneau, Pascal Bardeau, Daniel L. Coutu, Christian M. Beauséjour

**Affiliations:** 1 Centre de recherche du CHU Ste-Justine, Montréal, Québec, Canada; 2 Département de pharmacologie Université de Montréal, Montréal, Québec, Canada; 3 Stem Cell Dynamics Research Unit, Helmholtz Center Munich - German Research Center for Environmental Health, Munich, Germany; McGill University, Canada

## Abstract

DNA damage can lead to the induction of cellular senescence. In particular, we showed that exposure to ionizing radiation (IR) leads to the senescence of bone marrow-derived multipotent stromal cells (MSC) and osteoblast-like stromal cells (OB–SC), a phenotype associated with bone loss. The mechanism by which IR leads to bone dysfunction is not fully understood. One possibility involves that DNA damage-induced senescence limits the regeneration of bone progenitor cells. Another possibility entails that bone dysfunction arises from the inability of accumulating senescent cells to fulfill their physiological function. Indeed, we show here that exposure to IR prevented the differentiation and mineralization functions of MSC, an effect we found was limited to this population as more differentiated OB–SC could still form mineralize nodules. This is in contrast to adipogenesis, which was inhibited in both IR-induced senescent MSC and 3T3-L1 pre-adipocytes. Furthermore, we demonstrate that IR-induced loss of osteogenic potential in MSC was p53-dependent, a phenotype that correlates with the inability to upregulate key osteogenic transcription factors. These results are the first to demonstrate that senescence impacts osteogenesis in a cell type dependent manner and suggest that the accumulation of senescent osteoblasts is unlikely to significantly contribute to bone dysfunction in a cell autonomous manner.

## Introduction

Exposure to ionizing radiation (IR) is a major risk factor for the development of long-term side effects [1,2]. In particular, significant reduction in bone mineral density is observed several years following bone marrow transplantation, a procedure that often requires total body irradiation [3–5]. Reduced bone density and increased risk of fractures are also associated with age [6]. One hypothesis is that induction of cellular senescence following exposure to IR or during aging contributes to bone dysfunction.

Senescent cells are metabolically active but have permanently loss the ability to divide [7,8]. Cells can become senescent in response to various DNA damaging events such as telomere dysfunction, oncogenic mutations and exposure to chemoradiotherapy [9]. In opposition to apoptotic cells that are rapidly eliminated, senescent cells can accumulate in tissues [10,11]. Their accumulation along with the senescence associated secretion phenotype (SASP) is believed to have a detrimental impact on tissues homeostasis [12–14]. For example, systemic variations in various hormones and cytokines within the bone marrow microenvironment may negatively impact the regeneration of bone in a cell non-autonomous manner [15,16]. Alternatively, senescence could directly interfere with osteogenesis by preventing the proliferation and function of bone marrow multipotent stromal cells (MSC) which have the ability to differentiate in various cell populations including osteoblasts and adipocytes [17–19].

Induction of senescence is controlled primarily by the p53 and the retinoblastoma (pRb/p16^INK4a^) pathways [9,20]. The expression of p16^INK4a^ and the presence of unresolved DNA damage foci are arguably the most reliable in vivo senescent markers [21–23]. Both pathways are closely linked to bone homeostasis. For example, pRb was shown to be directly involved in the differentiation of bone progenitor cells by its ability to bind and activate key osteogenic regulators such as Runx2 [24,25]. pRb can also suppress adipogenic differentiation through its action on the peroxisome proliferator-activated receptor γ subunit (PPARγ) [24]. Similarly, downregulation of p53, or its downstream regulator p21^CIP1/Waf1^, was shown to enhance the proliferation and osteogenic differentiation potential of murine stromal cells [26–28]. Increased expression of the osteogenic transcriptional regulators Runx2 and Osterix (Osx) was identified has the probable mechanism underlying the control of osteogenesis by p53.

We recently showed that bone marrow-derived multipotent MSC and more differentiated osteoblast-like stromal cell (OB–SC) populations expressed markers of senescence following exposure of mice to IR [29]. We also observed an increase in the expression of osteoblast-related genes in irradiated MSC, a phenotype that correlated with the incapacity to fully maintain this population long term after mice were irradiated [29]. These observations led us to ask whether IR-induced senescence has a similar impact on the functionality of MSC and OB–SC cell populations. In the present study we provide evidences that IR-induced senescence interfere with osteogenesis, en effect we found was limited to bone progenitor cells and dependent on p53.

## Results

### Exposure to IR induces cellular senescence of stromal progenitor and committed cell lineages

**Figure 1:**
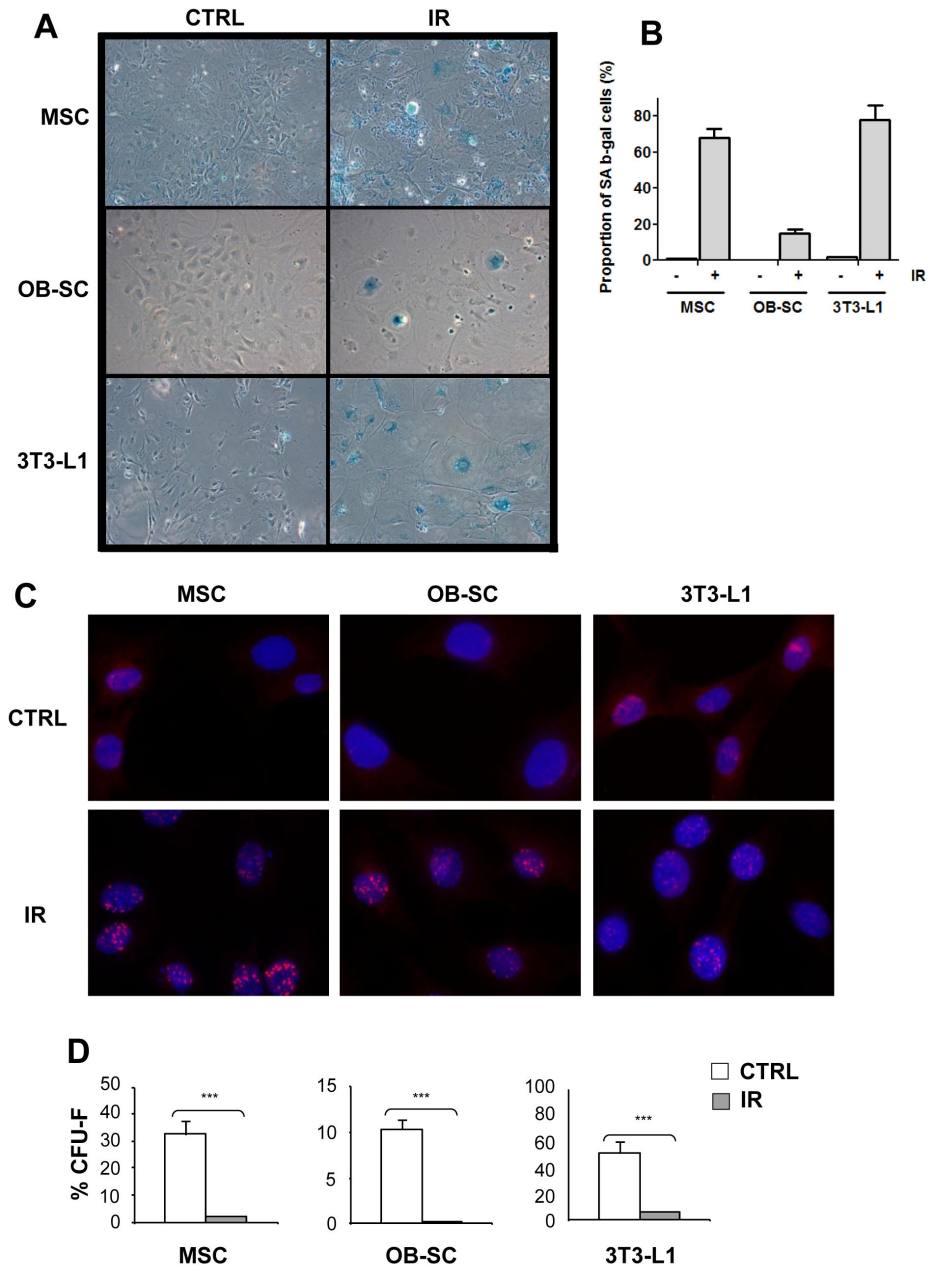
Senescence of multipotent and committed stromal lineages following exposure to IR. (**A**) Murine bone marrow-derived multipotent stromal cells (MSC), osteoblasts (OB–SC) and pre-adiopocytes (3T3-L1) were exposed (IR) or not (CTRL) to 10 Gy IR and 7 days later stained for the expression of the senescence-associated β-galactosidase (SAβ-gal). (**B**) Quantification of the proportion of SAβ-gal positive cells in each population. (**C**) Sustained activation of the DNA damage response in stromal populations was measured by staining for the presence of 53BP1 DNA damage foci (in red) one week post exposure to IR. Nuclei were counterstained with DAPI. (**D**) The proliferation capacity of MSC, OB–SC and 3T3-L1 cell population was determined using a CFU assay one week post-exposure or not to IR. Mean ± standard error of at least three individual experiments is shown. p values were obtained by performing a Student’s t-test.

Whether cells undergo cellular senescence or apoptosis in response to IR is cell type specific and needs to be determined. Bone homeostasis is believed to rely, at least in part, on bone marrow-derived MSC through their ability to differentiate in osteoblasts and/or adipocytes. Using defined purification procedures [29], we isolated multipotent MSC and more committed osteoblasts (OB–SC) and exposed these populations, along with the pre-adipocyte 3T3-L1 cell line, to 10 Gy IR. While irradiated cell populations showed no sign of apoptosis (data not shown), they all expressed markers of senescence (Figure 1). Indeed, irradiated populations displayed an enlarged cytoplasm and a flattened appearance, two morphological characteristics of senescent cells. Moreover, with the exception of the OB–SC population, the majority of cells stained positive for the senescence-associated β-galactosidase (SAβ-gal) (Figure 1A and 1B). Persistent DNA damage, as detected by 53BP1 foci, was also observed in all senescent populations, a phenotype almost completely absent in control non irradiated cell populations (Figure 1B). Finally, the expected loss in the proliferation potential of all three irradiated cell populations was confirmed using a colony forming unit (CFU) assay (Figure 1C). These results demonstrate that bone stromal progenitor cells and more differentiated lineages undergo senescence in response to IR-induced DNA damage.

### Induction of senescence interferes with the adipogeneic differentiation potential

**Figure 2:**
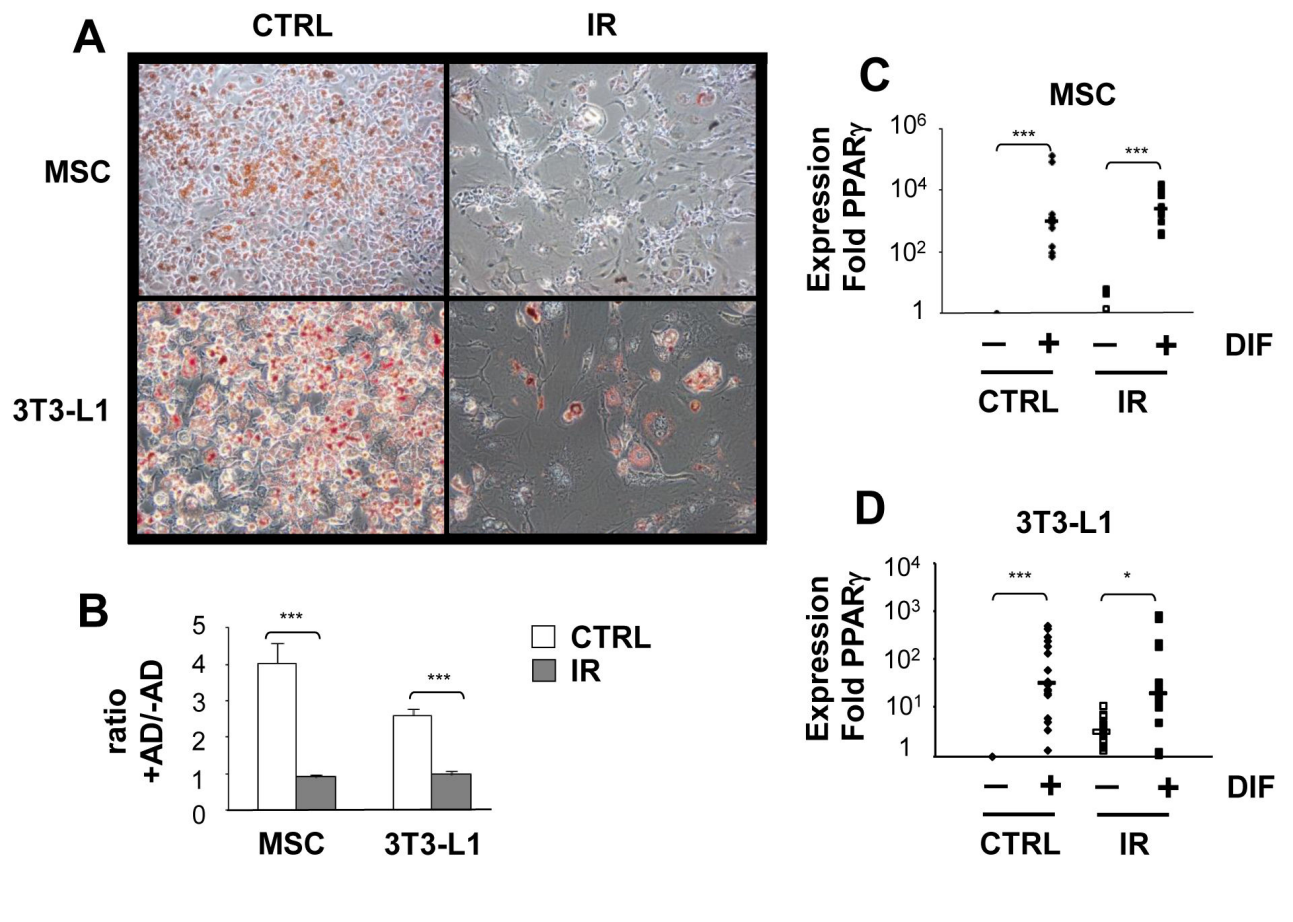
Exposure to IR abrogates adipogenesis independently of the stromal lineage potential. (**A**) MSC and 3T3-L1 cell populations were exposed (IR) or not (CTRL) to 10 Gy IR and one week later placed in adipogenic differentiation media. Representative photographs showing lipid accumulation stained with Oil Red O is shown for each population. (**B**) Quantification of lipid accumulation was determined by the extraction of Oil Red O staining and detection by spectrophotometry. (**C**–**D**) Expression of PPARγ was determined by quantitative real-time PCR using RNA extracted from control and IR-induced senescent MSC and 3T3-L1 populations cultured or not in adipogenic differentiation media. Mean ± standard error of at least three individual experiments is shown. p values were obtained by performing a Student’s t-test. *: *p* value < 0.05.

It is well known there is an increased adipocyte content in the bone marrow with age or following exposure to IR. We therefore investigated whether IR-induced senescence could skew the differentiation potential of multipotent MSC toward the adipogenic lineage. As such, control and one week post-irradiation MSC populations were cultured under adipogenic conditions and their differentiation measured. While control cultures of MSC differentiated into adipocytes, irradiated cell populations failed to do so (Figure 2A). This loss in the adipogenic potential was not unique to MSC progenitors as senescent 3T3-L1 pre-adipocytes also failed to differentiate under the same conditions (Figure 2A). Differentiation was evaluated by the ability to accumulate lipids as showed by quantification of Oil Red O staining (Figure 2B). Failure to differentiate into the adipogenic lineage was not due to the incapacity to upregulate the key adipogenic transcription factor PPARγ which we showed the expression was increased 100-1000 fold in both senescent MSC and 3T3-L1 populations cultured in adipogenic conditions. These results suggest that failure of IR-induced senescent cells to undergo adipogenic differentiation is independent of the multilineage potential and on the ability to activate PPARγ.

### Inhibition of the osteogenic differentiation potential during senescence is restricted to stromal progenitor cells

**Figure 3:**
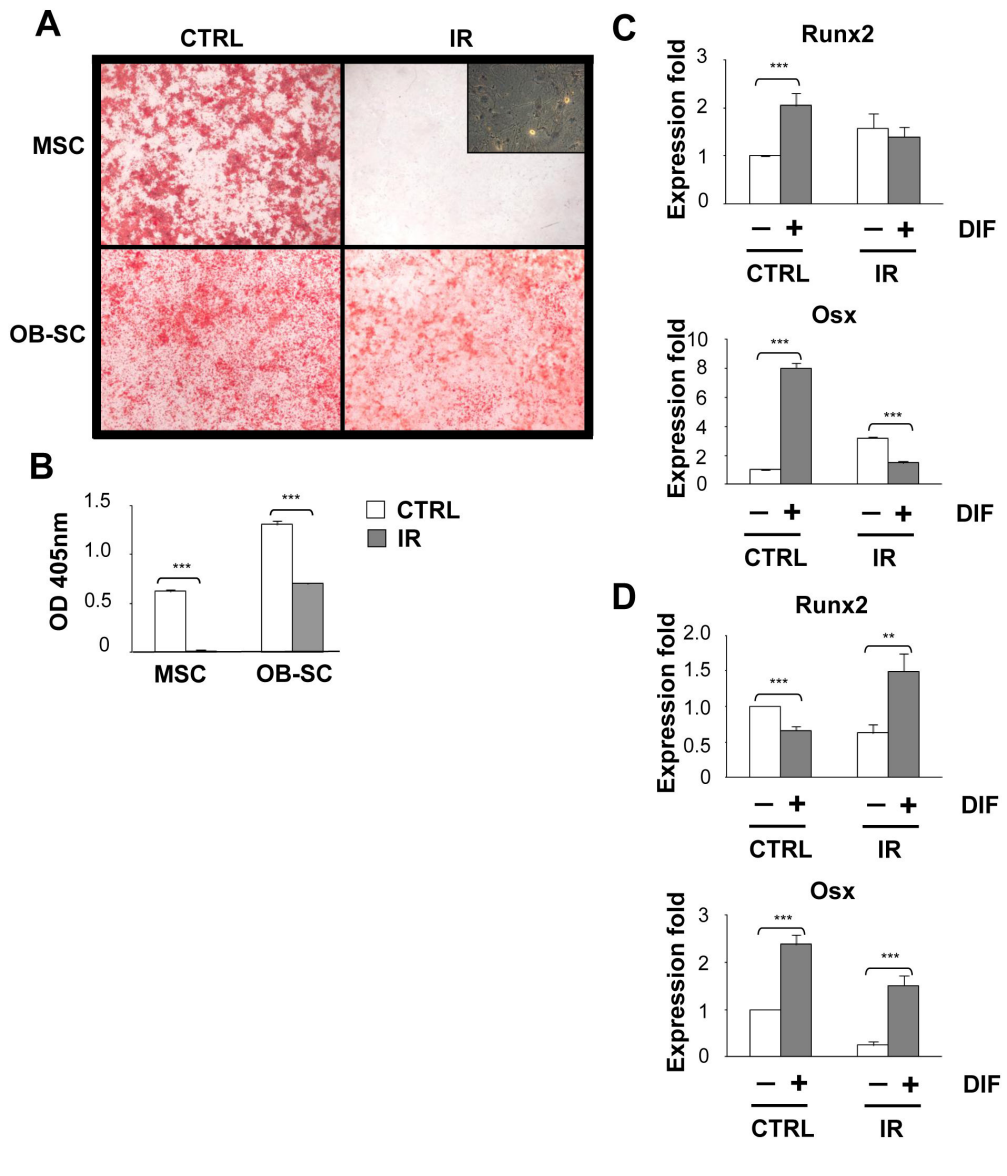
Abrogation of osteogenic differentiation potential following irradiation is limited to stromal progenitor cells. (**A**) MSC and osteoblasts (OB–SC) were exposed (IR) or not (CTRL) to 10 Gy IR and one week later placed in osteogenic differentiation media for 14 to 21 days. Representative photographs showing mineralization nodules accumulation stained with Alizarin Red S is shown for each population. Scale bar: 2mm. Phase contrast photograph showing the presence of senescent MSC in absence of mineralization is also shown. (**B**) Quantification of mineralization was determined by the extraction of Alizarin Red S and detection by spectrophotometry. (**C** and **D**) Expression of Runx2 and Osx was determined by quantitative real-time PCR using RNA extracted from control and IR-induced senescent MSC and OB–SC populations cultured or not in osteogenic differentiation media. Mean ± standard error; *: *p* value < 0.05.

Total body irradiation leads to a net bone loss, an effect that coincides with the presence of senescent cells and a diminution in the absolute number of stromal and hematopoietic stem/progenitor cells [4,29]. However, whether the bone mineralization potential of MSCs and osteoblasts is differently affected by the senescent phenotype is currently unknown. Therefore, we exposed MSC and OB–SC populations to 10 Gy IR in vitro and investigated their ability to mineralize and upregulate key osteogenic transcription factors when placed in differentiation media. As we observed for adipogenesis, we found the osteogenic potential of MSC progenitors to be severely abrogated post IR, as no mineralized nodules were observed in senescent cultures when compared to control non-irradiated populations (Figure 3A). This contrasts to what we observed in the OB–SC population which surprisingly could still efficiently form mineralized nodules after irradiation, although the process was reduced compared to non-irradiated cultures. Solubilisation and quantification of Alizarin Red by spectrometry allowed us to precisely quantify the mineralization process (Figure 3B).

We hypothesized that the failure of senescent MSC to mineralize could be due to their inability to upregulate Runx2 and Osx, two transcription factors that control the commitment of MSC into the osteogenic lineage and the late stages of osteogenesis respectively [26,28]. Indeed, we found that senescent MSC failed to fully upregulate Osx, and to a lesser extent Runx2, when cultured under osteogenic conditions compared to control non-irradiated MSC (Figure 3C). In contrast, the expression of Osx and Runx2 was increased in senescent OB–SC placed under differentiation conditions (Figure 3D). Not surprisingly, the fold increase in the expression of Runx2 and Osx was less pronounced in stimulated OB–SC compared to MSC, which is consistent with the high expression levels expected for these factors in already committed cells.

**Figure 4:**
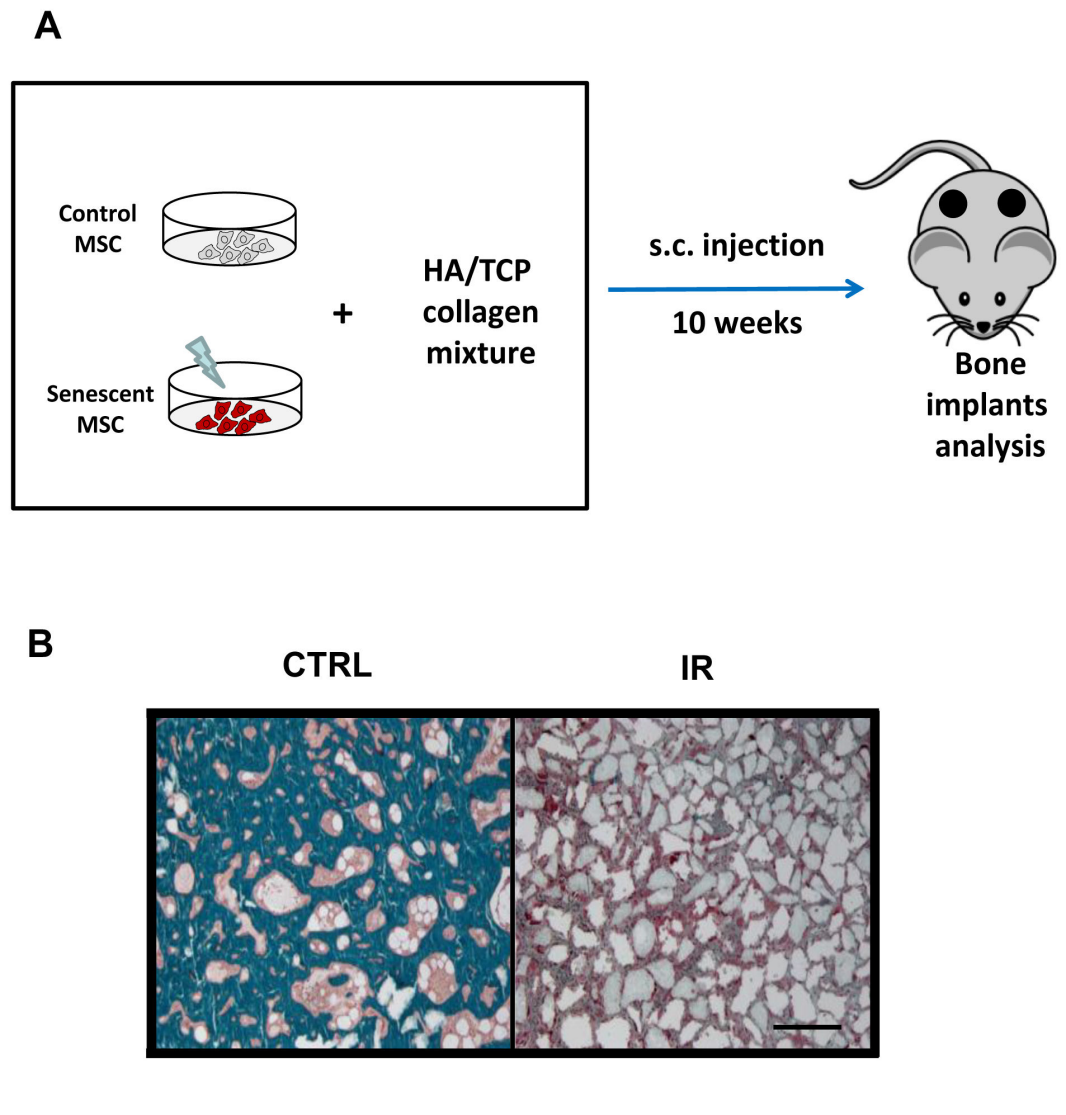
IR-induced senescent MSC failed to generate bone in vivo. (**A**) Schematic of the experiment. Control or IR-induced senescent MSC were mixed with HA/TCP particles along with collagen and injected subcutaneously to the flank of mice. 10 weeks post injection, implants were retrieved from the animals, embedded in plastic, sectioned and stained with Goldner’s trichrome to detect bone formation. (B) Representative images from n= 6 implants per group showing mineralization (Goldner’s trichrome in green) from control or IR-induced senescent MSC. Implants were counterstained with hematoxylin eosin. Scale bar: 300µm.

Finally, because the osteogenic differentiation condition established in vitro may not adequately reflect in vivo conditions, one may argue that loss of mineralization potential observed in senescent MSC is a culture artifact and that senescent MSC may still be able to differentiate in vivo. To address this important question, we used a heterotopic bone formation model in which MSC, when mixed with hydroxyapatite/tricalcium phosphate particles and collagen can form bone subcutaneously in mice (Figure 4A). Using this model, we observed robust heterotopic bone formation in MSC implants as shown by Goldner’s Trichrome staining (Figure 4B). In contrast, senescent MSC were unable to mineralize bone in vivo, indicating that the loss in ostegenic differentiation potential following exposure to IR likely takes place under physiological conditions as well. Importantly, lack of mineralization in vivo was not due to the death/clearance of senescent MSC as we have injected IR-induced senescent MSC subcutaneously in mice before and never observed reduced viability of these cells compared to control non-irradiated MSC [30]. Together, these results suggest that the net bone loss observed following exposure to IR may not only arise as the consequence of the depletion of bone progenitor cells but also as a result of impaired functionality from DNA damaged MSC.

### Loss of osteogenic potential in senescent MSC is mediated by p53

**Figure 5:**
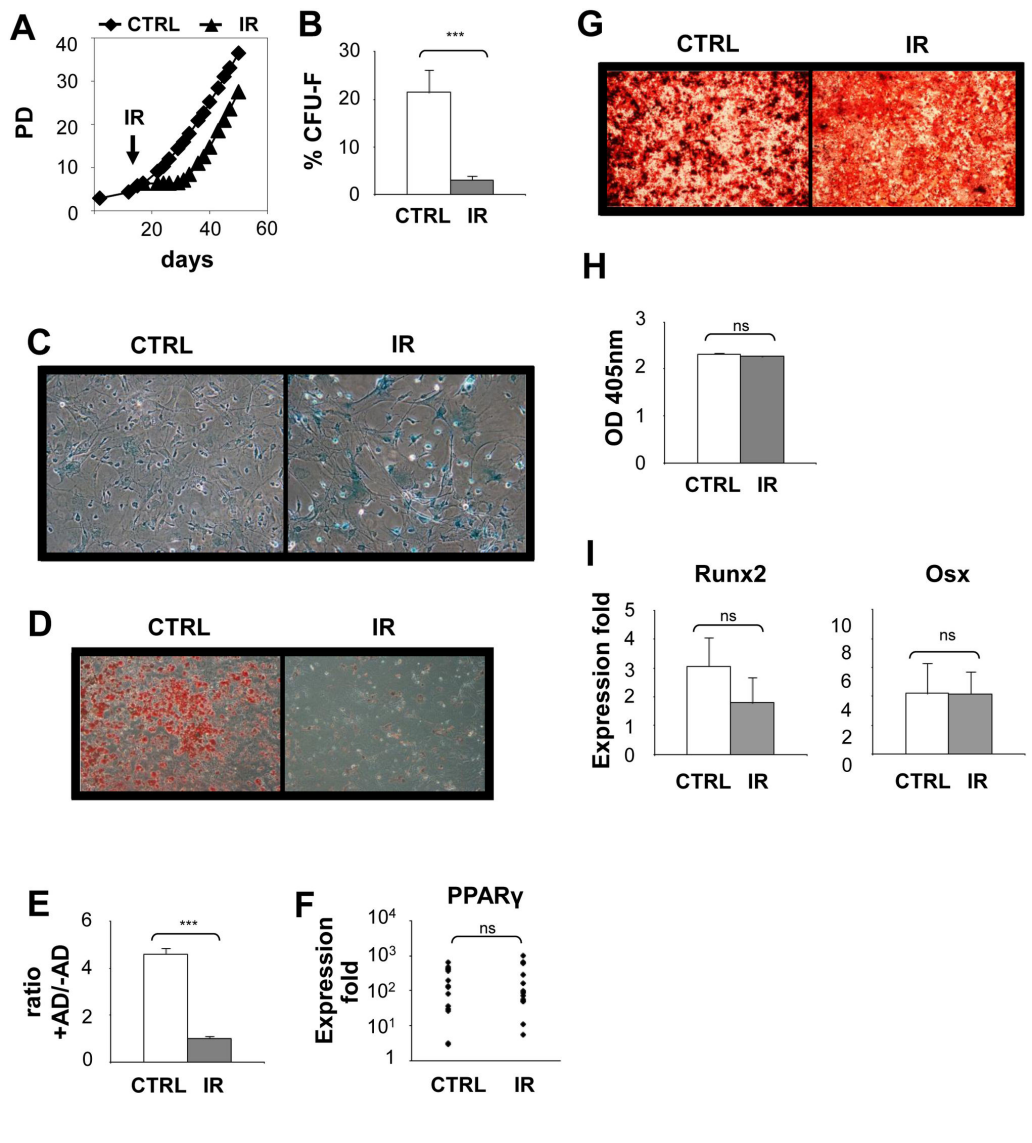
Loss of osteogenic but not adipogenic potential in senescent MSC is p53 dependent. (**A**) MSC derived from p53 knockout mice (MSC-p53KO) were exposed (IR) or not (CTRL) to 10 Gy IR and one week later stained for the expression of SAβ-gal activity. (**B**) The proliferation capacity of MSC-p53KO was determined using a CFU assay one week post-exposure or not to IR. (**C**) One week post exposure or not to IR, MSC-p53KO were placed in adipogenic differentiation media for 7 to 14 days. Representative photographs showing lipid accumulation stained with Oil Red O is shown. Scale bar: 200µm. (**D**) Quantification of lipid accumulation has determined by the extraction of Oil Red O staining and detection by spectrophotometry. (**E**) Expression of PPARγ was determined by quantitative real-time PCR using RNA extracted from control and IR-induced senescent MSC-p53KO cultured in adipogenic differentiation media. (**F**) One week post exposure or not to IR, MSC-p53KO were placed in osteogenic differentiation media for 14 to 21 days. Representative photographs showing mineralization nodules accumulation stained with Alizarin Red S is shown. (**G**) Quantification of mineralization was determined by the extraction of Alizarin Red S staining and detection by spectrophotometry. (**H**) Expression of Runx2 and Osx was determined by quantitative real-time PCR using RNA extracted from control and IR-induced senescent MSC-p53KO populations placed in osteogenic differentiation media. Mean ± standard error of at least 3 individual experiments is shown; *: *p* value < 0.05.

A known transcriptional regulator for adipogenesis and osteogenesis is the tumor suppressor p53. Furthermore, p53 was shown to regulate cellular senescence in response to several biological processes, including exposure to IR. We therefore investigated whether p53 activation, in response to IR-induced DNA damage, was involved in the blockage of osteogeneic and adipogenic differentiation during senescence. MSC were derived from the bone marrow of p53 knockout mice and exposed to IR as previously done. We found that MSC deficient for p53 could still adopt a senescent morphology, expressed the SAβ-gal marker, and lose their proliferation capacity as measured by a CFU formation assay (Figure 5A–C). However, it should be noted that despite the fact that p53 deficient MSC undergo senescence in response to IR, clones escaped senescence in the weeks following irradiation. As such, the differentiation potential of p53 deficient MSC was evaluated in the first week post exposure to IR, a time at which most if not all irradiated cells were senescent. Under these conditions, we found the absence of p53 was insufficient to restore the differentiation of senescent MSC into adipocyte, despite robust expression of PPARγ (Figure 5D–F). Conversely, p53 deficient senescent MSC were able to form numerous mineralized nodules in vitro at levels similar to control non-irradiated cultures (Figure 5G and H). Moreover, in accordance with their ability to mineralize, senescent MSC deficient for p53 could efficiently upregulate Osx and Runx2 when placed in differentiation media (Figure 5I). These results suggest that the osteogenic but not the adipogenic differentiation potential of MSC is regulated by p53 in senescent cells.

## Discussion

Inadequate bone formation following exposure to IR is believed to arise as the consequence of both extrinsic and intrinsic factors [31]. In this study we focused on cell autonomous defects, particularly the impact that IR-induced senescence has on osteoblast (OB–SC) and bone progenitors (MSC) functions. We found that while the proliferation potential of both MSC and OB–SC populations is severely compromised post IR, only senescent MSC were functionally affected as defined by their inability to form mineralize nodules and to express key osteogenic transcription factors after stimulation. This is in contrast to the adipogenic differentiation potential which was inhibited in both MSC and more committed 3T3-L1 senescent cells. Our findings suggest that reduced bone mineral density observed following exposure to IR is likely independent on the ability of senescent osteoblasts to mineralize bone. Instead, this reinforces the hypothesis that diminished osteogenesis following exposure to IR is more likely to be caused, at least partially, from impaired bone regeneration. Supporting this hypothesis, we previously showed that exposure of mice to IR leads to the senescence of bone marrow-derived MSC and to the depletion of this population as early as 8 weeks post IR [29].

Our data also revealed that the failure of senescent MSC to undergo osteogenic differentiation was dependent on p53 and that it coincided with the inability to upregulate the transcription factor Osx. This is in opposition to what we observed in more committed OB–SC where upregulation of Osx occurred in senescent cells. These observations suggest that activation of p53 during senescence prevents osteogenic differentiation in a cell type dependent manner. These results are in accordance with previous studies showing that p53 represses the transcription of Osx and that knockdown of p53 favors osteogenic differentiation [26,28]. However, the mechanism by which p53 restricts the differentiation of MSC in response to IR likely involves one or several factors other than Osx, as complementation studies where Osx was constitutively expressed failed to restore the differentiation potential of senescent MSC (Figure S1).

Our results are in agreement with the data from Mehrara and colleagues who showed that increased expression of p21^CIP1/Waf1^ following exposure to IR interferes with the adipogenic and osteogenic differentiation of MSC [32]. However, the mechanism linking p53/ p21^CIP1/Waf1^ to osteogenesis remains to be fully elucidated given we found that increased expression of p21^CIP1/Waf1^, which also occurs in IR-induced senescent OB–SC (data not shown), does not interfere with their capacity to mineralize. One possibility may include a higher basal expression level of key ostegenic transcription factors in more committed OB–SC compared to MSC, making these cells less sensitive to p53/ p21^CIP1/Waf1^ transcriptional repression during senescence.

Like p21^CIP1/Waf1^, p16^INK4a^ is another senescent inducer which the expression in irradiated MSC populations could have played a role in controlling osteogenesis [29]. Indeed, increased expression of p16^INK4a^ in known to induce cell cycle arrest by preventing the phosphorylation of pRb. Given pRb was shown to act as a direct transcriptional coactivator of Runx2, it is possible that the expression of p16^INK4a^ interferes, probably in an indirect manner, with the transcriptional functions of pRb. In support of this hypothesis, we found that MSC isolated from Ink4a/Arf knockout mice had an increased mineralization potential compared to wild type mice (data not shown). Similarly, deletion of Ink4a/Arf also strongly promoted their differentiation into adipocytes (data not shown), a phenotype that may be explained by the action of p16^INK4a^ on the cyclin-dependent kinase 4 and PPARγ activation [33]. Hence, as we observed for p21^CIP1/Waf1^, expression of p16^INK4a^ is unlikely to explain the cell type dependent effect of senescence on osteogenesis given that both MSC and OB–SC expressed p16^INK4a^ following exposure to IR [29]

Finally, despite the evidences presented here that IR-induced bone loss is cell autonomous, the influence an irradiated bone microenvironment may have on bone homeostasis remains largely unknown. This is partly due to the difficulty in measuring endogenous bone formation based only on extrinsic systemic factors. We have tried addressing this question by measuring the impact of an irradiated systemic environment using the heterotopic bone formation model in which the hosts (not the MSC) were previously exposed or not to irradiation. Using this model, we did not observe a reduced bone formation in implants collected from mice exposed to total body irradiation 8 weeks prior to the injection of MSC (Carbonneau et al. in preparation). This suggests that the systemic environment from an irradiated host has little impact on the bone formation rate when compared to a non-irradiated host. However, one limitation from this model is that the microenvironment found subcutaneously may differ from the one found in bones. As such, we cannot rule out a role for extrinsic systemic factors in bone formation following IR, especially that it has been shown that an inflammatory microenvironment favors bone loss through increased osteoclastogenesis in telomerase or DNA repair-deficient mice [34,35]. This comparison is particularly relevant given that senescence induced by short telomeres is mechanistically similar to IR-induced senescence and that they both involve a p53 mediated DNA damage response [36].

Overall, our results are the first to demonstrate that senescent cells can maintain normal osteogenic functions in a cell type dependent manner. Similarities in the role of p53 in inducing senescence in mouse and human cells following DNA damage suggest that the impact of irradiation on ostegenesis is likely to be similar in patients. Given the growing body of evidence showing the deleterious physiological effect of senescent cell accumulation [14], a better understanding of the impact of senescence on bone homeostasis will help in the design of better treatments to alleviate bone dysfunction and other disabilities often observed in aged or irradiated patients.

## Materials and Methods

### Cell isolation

All cells were grown in α-MEM (Multicell, Woonsocket, RI, USA) supplemented with 10mM L-glutamine (Invitrogen, Burlington, Canada) and 10% fetal calf serum (Multicell) in a humidified chamber at 37°C and 6% CO_2_. Bone marrow-derived mensenchymal stromal cells (MSC) and bone-derived osteoblast-like stromal cells (OB–SC) were derived from bone marrow and bone chips respectively, and isolated based on their property to attach to polystyrene after having been cultured for one week and their phenotype defined by flow cytometry, as described previously [29]. Of note, despite with observed no major differences on the impact of IR on the differentiation potential of clonal and non-clonal MSC populations, results from a clonal population are presented in this manuscript in order to avoid heterogeneity in multilineage potential. MSC populations deficient for p53 were isolated from the bone marrow of p53 knockout mice using the above described procedure.

### Irradiation procedure and cell plating

Cell cultures at 80% confluence were irradiated at 10 Grays using a Gammacell 220 and cobalt-60 as a source. Five to seven-day post-irradiation cells were trypsinized, serially diluted, plated in 6-well plates, and maintained at 37°C 6% CO_2_ 3% O_2_ for seven days. Colonies were then fixed and stained with 0.5% methylene blue in 50% methanol for CFU-F counting. Alternatively, five to seven-day post-irradiation cells were plated in 12-well plate for differentiation assay. One to two days post-plating, cultures were exposed to adipogenic or osteogenic conditions in a humidified chamber at 37°C and 6% CO_2_. Fractions of control and irradiated p53^-/-^ MSC populations were plated in 25cm^2^ flaks for cell growth assays after irradiation. Population doubling of the control and irradiated cultures was calculated using the formula log_10_(final number of cells) – log_10_ (initial number of cells plated)/0.301.

### Adipogenic assay

Confluent cells were cultured with complete medium supplemented with 1 µM dexamethasone, 10µg/ml insulin, 50µM indomethacine, and 0.5µM isobutyl methyxanine (all reagents from Sigma Aldrich, Oakville, Canada). Media change was done every other day. After seven to 14 days, cells were fixed in 3.7% formaldehyde, followed by staining with a solution of 0.21% Oil Red O in 60% isopropanol (all reagents from Sigma Aldrich). Cultures were photographed with an inverted microscope ECLIPSE TE300 (Nikon, Mississauga, Canada). Oil Red O staining was extracted with 100% isopropanol, followed by optical density measurement at 500nm.

### Osteogenic assay

Confluent cells were cultured under osteogenic conditions for 14 to 21 days with 100nM dexamethasone, 10mM beta-glycerophosphate, 50µM vitamin C, and 50nM vitamin D_3_ (all products from Sigma Aldrich) in complete medium. Media change was done every other day. Mineralized nodules were stained with 0.5% Alizarin Red S solution pH 4.2 (Sigma Aldrich). Photographs were taken with a stereomicroscope Leica MZ FLIII (Leica, Concord, Canada). Alizarin Red S was extracted and quantified using a previously described method [37].

### Senescence-associated β-galactosidase staining

Control and five to 10 days post-irradiation cells seeded on glass slides were fixed in 3.7% formaldehyde and rinsed in PBS. Fixed cells were stained with the following buffer: 40mM citric acid/phosphate pH 5.0 supplemented with 5mM potassium ferricyanide, 5mM potassium ferrocyanide, 150mM NaCl, 2mM MgCl2 and 1mg/ml X-gal (all reagents from Sigma, except X-gal from Invitrogen, Burlington, ON, Canada). Slides were incubated for 16 hours at 37°C in a humidified chamber, followed by rinses in PBS and milli-Q water. Slides were mounted and photographed using an inverted microscope ECLIPSE TE300 (Nikon).

### Immunostaining

Control and five to 10 days post-irradiation cells seeded on glass slides were fixed in 3.7% formaldehyde and rinsed in PBS. Cells were permeabilized with 0.5% Triton in PBS for five minutes, followed by three rinses in PBS. Blocking was done for one hour at room temperature with 5% goat serum and 1% bovine albumin in PBS. Staining of 53BP1 protein was done by incubation with a rabbit anti-mouse antibody (clone 304, Novus Biologicals, Oakville, ON, Canada) in 2.5% goat serum 1% bovine albumin in PBS for 16 hours at 4°C. A goat anti-rabbit antibody (Invitrogen) coupled to Alexa Fluor594 diluted in 1% bovine albumin in PBS was used to reveal 53BP1. Nuclei were stained with DAPI. Slides were mounted and photographed with a BX51 upright microscope (Olympus, Richmond Hill, Ontario, Canada).

### Quantitative RT PCR

Total RNA extraction from cultured cells was done with the RNeasy mini extraction kit (Qiagen, Toronto, Canada). Five hundred nanograms of total RNA were subjected to reverse transcription reaction using the QuantiTect Reverse Transcription kit (Qiagen). Quantitative PCR was done with the SensiMix SYBR Green low ROX kit (Bioline, Medicorp, Montreal, Canada). Primer sequences are the following: Osterix forward 5’- TCTCCATCTGCCTGACTCCT-3’ and reverse 5’- AGCGTATGGCTTCTTTGTGC-3’; PPARγ forward 5’-CTCCTGTTGACCCAGAGCAT-3’ and reverse 5’-AATGCGAGTGGTCTTCCATC-3’; Runx2 forward 5’- GCCGGGAATGATGAGAACTA-3’ and reverse 5’- GGACCGTCCACTGTCACTTT-3’. Amplification was done with initial denaturation of 15 minutes at 95°C, followed by 40 cycles of 15sec at 95°C, 15sec at 60°C and 30sec at 72°C, and a final denaturation step of 1min at 95°C, 30sec at 55°C, 30sec at 95°C. Results are expressed with the 2^-ΔΔCt^ method [38].

### Heterotopic implants

Control and five day post-irradiation MSC were trypsinised and incubated with hydroxyapatite/tricalcium phosphate (HA-TCP) particles at a ratio of 2x10^6^ cells/80mg of particles for each implant. Cells and particles were incubated at 37°C for 2hrs under constant slow agitation. A collagen gel at pH 7.0 made of 0.0065mg/ml collagen type I (BD, Mississauga, Ontario, Canada), 1.32mM DTT (Sigma), 6.6mM CaCl_2_ (Sigma), 0.32U/ml transglutaminase (Sigma) was aliquoted at 1.47mg of collagen type I per 2x10^6^ cells/80mg particles. Mixtures of cells, particles and collagen gel were kept on ice until implantation. C57Bl/6 mice from Jackson Laboratories (Bar Harbor, ME) were anesthetized and a small incision was made on the back and the skin lifted from connective tissues. Cell mixture was pipetted into the cavity. Surgical clips were used to close the wound and removed at one week post-surgery. Mice were sacrificed by carbon dioxide inhalation 10 weeks later and implants were excised and fixed in formalin (Sigma). Implants were embedded into methylmetacrylate for cutting. Slides were stained with Goldner’s trichrome and photographed using a dissection microscope M205 FA (Leica, Concord, ON, Canada). All *in vivo* manipulations were approved by the Comité Institutionnel des Bonnes Pratiques Animales en Recherche of CHU Ste-Justine (protocol number 421).

## Supporting Information

Figure S1 **Forced expression of Osx does not rescue IR-induced blockade of osteogenesis in senescent MSC.** MSC were transduced with GFP or Osx expressing lentiviral vectors and gene-modified cells selected with puromycine. Cells were then expanded for a few population doubling and forced expression of Osx determined by quantitative real-time PCR (A). In parallel, cell populations were exposed or not to IR and cultured under osteogenic conditions for 14 days and the ability to formed mineralized nodules in vitro evaluated (B). Mean ± standard error of at least three individual experiments is shown; *: *p* value < 0.05.
(TIF)
