# Supplementary figures and images for: Loss of the Osteogenic Differentiation Potential during Senescence Is Limited to Bone Progenitor Cells and Is Dependent on p53
Source: PLoS One. 2013 Aug 29;8(8):e73206. doi: 10.1371/journal.pone.0073206 (PMC3756945; doi:10.1371/journal.pone.0073206)

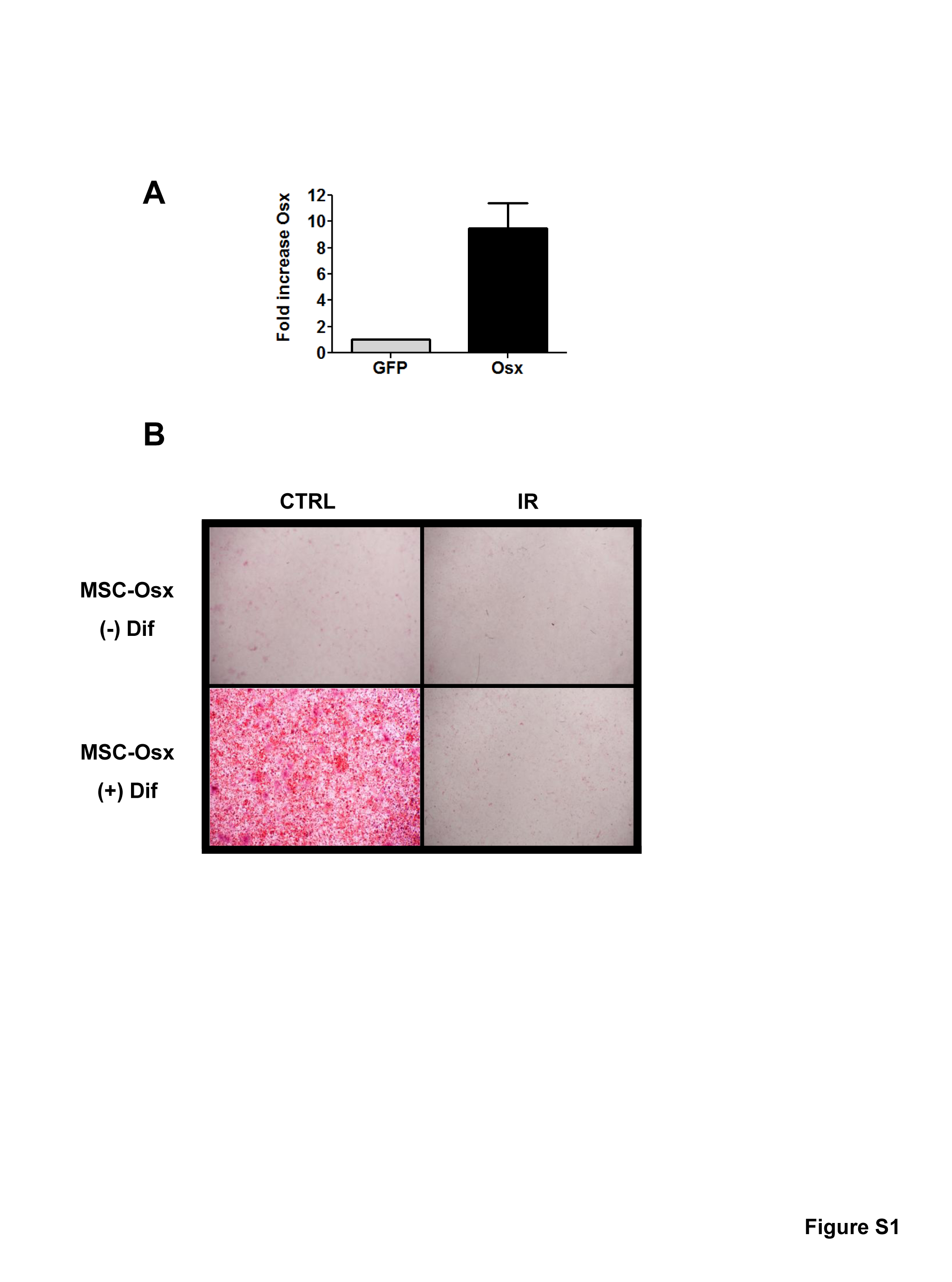

Supplement: Figure S1 — Forced expression of Osx does not rescue IR-induced blockade of osteogenesis in senescent MSC. MSC were transduced with GFP or Osx expressing lentiviral vectors and gene-modified cells selected with puromycine. Cells were then expanded for a few population doubling and forced expression of Osx determined by quantitative real-time PCR (A). In parallel, cell populations were exposed or not to IR and cultured under osteogenic conditions for 14 days and the ability to formed mineralized nodules in vitro evaluated (B). Mean ± standard error of at least three individual experiments is shown; *: p value < 0.05. (TIF) [file pone.0073206.s001.tif]
